# *In Situ* Spectral Kinetics of Cr(VI) Reduction by *c*-Type Cytochromes in A Suspension of Living *Shewanella putrefaciens* 200

**DOI:** 10.1038/srep29592

**Published:** 2016-07-11

**Authors:** Tongxu Liu, Xiaomin Li, Fangbai Li, Rui Han, Yundang Wu, Xiu Yuan, Ying Wang

**Affiliations:** 1 Guangdong Key Laboratory of Agricultural Environment Pollution Integrated Control, Guangdong Institute of Eco-Environmental and Soil Sciences, Guangzhou, 510650 P. R. China; 2 School of Civil and Environmental Engineering, University of New South Wales, Sydney, NSW, 2052 Australia

## Abstract

Although *c*-type cytochromes (*c*-Cyts) mediating metal reduction have been mainly investigated with *in vitro* purified proteins of dissimilatory metal reducing bacteria, the *in vivo* behavior of *c*-Cyts is still unclear given the difficulty in measuring the proteins of intact cells. Here, *c*-Cyts in living *Shewanella putrefaciens* 200 (SP200) was successfully quantified using diffuse-transmission UV/Vis spectroscopy due to the strong absorbance of hemes, and the *in situ* spectral kinetics of Cr(VI) reduction by *c*-Cyts were examined over time. The reduced product Cr(III) observed on the cell surface may play a role in inhibiting the Cr(VI) reduction and reducing the cell numbers with high concentrations (>200 μM) of Cr(VI) evidenced by the 16S rRNA analysis. A brief kinetic model was established with two predominant reactions, redox transformation of *c*-Cyts and Cr(VI) reduction by reduced *c*-Cyts, but the fitting curves were not well-matched with *c*-Cyts data. The Cr(III)-induced inhibitory effect to the cellular function of redox transformation of *c*-Cyts was then added to the model, resulting in substantially improved the model fitting. This study provides a case of directly examining the reaction properties of outer-membrane enzyme during microbial metal reduction processes under physiological conditions.

Dissimilatory metal reducing bacteria (DMRB) have a significant amount of *c*-type cytochromes (*c*-Cyts) in their outer membrane (OM)^1^. During anaerobic respiration, the *c*-Cyts mediate metal reduction from the cell surface to terminal electron acceptors (e.g., organics, electrodes, and metals)^2^,^3^, which is an important component of the biogeochemical cycling of metals and microbial activities in anoxic subsurface environments^4^,^5^. Over the last 20 years, many studies have examined *c*-Cyts of *Shewanella* or *Geobacter* using whole cells, lysates, cell fractions, and proteomic^6^,^7^,^8^,^9^. The reaction mechanism of reduced *c*-Cyts of *Geobacter* with various metals has also been studied^10^. In addition, a few investigations have focused on *in vitro* studies of purified OM *c*-Cyts from DMRB^11^,^12^,^13^,^14^ with reaction kinetics observed from the reaction between reduced *c*-Cyts and metals. However, a large discrepancy in redox properties was reported between purified proteins and protein complexes in a living cell because the highly reactive enzymes may readily be altered during purification^15^. As proteins embedded in a lipid membrane may function together as a whole in the intact cell, their properties should be influenced by a shift in the electron equilibrium^15^,^16^. Therefore, an *in vivo* study of the reaction between *c*-Cyts and metals will be valuable for comprehensively understanding the microbial metal reduction process.

Because the active center of *c*-Cyts, heme irons, have a large molar absorption coefficient, spectroscopic methods have been employed to investigate the *in vivo* properties^15^,^17^. Recently, some attempts have been made to investigate *c*-Cyts in living cell suspensions by employing spectral methods such as evanescent wave spectroscopy and surface-enhanced infrared absorption^18^. An *in situ* spectroscopic method was also used to study the reaction between Fe^3+^ and *c*-Cyts in intact *Leptospirillum ferrooxidans* under aerobic conditions^19^. UV/Vis absorption spectroscopy is useful to estimate bacterial numbers in suspension based on the absorption of cells; however, its application to other measurements is limited by the amount of scattered light from cell surfaces^15^. Fortunately, by using a diffuse-transmission (DT) mode, such spectral interference was not observed in the absorption spectra of multiheme *c*-Cyts in whole cells^15^. Recently, we also used DT-UV/Vis spectroscopy to directly measure the reduced or oxidized *c*-Cyts in a series of iron-/humic-reducing bacteria^20^,^21^,^22^, and the quantification of *c*-Cyts in intact cells has been made in terms of the hemes with well-known absorbance coefficients. Therefore, the *in situ* spectral kinetic analysis of the reactions in intact DMRB cells by DT-UV/Vis spectroscopy appears to be quite promising for the study of the microbial metal reduction process in a living cell system.

Because reductive transformation of Cr(VI) to Cr(III) is an established method to remediate Cr(VI) contamination^23^, and because Cr(VI) can be easily measured by spectrometric methods, the Cr(VI) reduction by *Shewanella putrefaciens* 200 (SP200) would be a suitable model reaction for investigating the kinetics between metal ions and *c*-Cyts in whole cells. While there are a tremendous amount of studies about microbial Cr(VI) reduction under the cellular levels from the aspects of kinetics, pathway, and intermediate products^8^,^9^,^10^, few focused on the reaction between Cr(VI) and *c*-Cyts in the intact cells due to the above mentioned limited methodology in measuring the apparent kinetic of *c*-Cyts. Kinetic modeling approach is a very powerful tool for elucidating the entire elementary reaction mechanism^24^, which would be very useful in illustrating the elementary reactions of microbial Cr(VI) reduction. However, previous studies of kinetic models were mainly focusing on homogeneous systems containing well-known molecules^25^, and the major challenge for applying such modeling approaches in microbial Cr(VI) reduction lies on an in-depth understanding of the molecular-reaction mechanisms between the critical enzymes and Cr(VI). Given the complexity of microbial system with many unknown species, a simplification of these mechanisms into some dominant reactions might be a good start for the application of modeling approaches in microbial Cr(VI) reduction. As the reaction between Cr(VI) and *c*-Cyts in the intact cells can be presented using a few very clear chemical reactions based on the previously-reported mechanisms, it will be very feasible to establish a brief model for describing the Cr(VI) reduction by *c*-Cyts in the intact cells.

To the best of our knowledge, there was no report examining the *in situ* reaction kinetics between Cr(VI) and *c*-Cyts in the intact cells, resulting in a poor understanding of the roles of *c*-Cyts in microbial metal reduction from the *in vivo* kinetics perspective^26^,^27^,^28^,^29^,^30^,^31^. In addition, the rate-determining steps and quantitative evaluations of each elementary reaction are still unclear, so a model approach in terms of the observed *in situ* kinetics as above and an interpretation of the elementary reactions might be very helpful for understanding the molecular-level reaction mechanisms between Cr(VI) and *c*-Cyts. Herein, the objectives of this study are as follows: (i) to quantify the *c*-Cyts in the living SP200 cell suspensions by DT-UV/Vis spectroscopy, (ii) to monitor *in situ* the changes of Cr(VI) and *c*-Cyts in the intact SP200 cells, and (iii) to quantitatively describe the *in vivo* reactions between Cr(VI) and *c*-Cyts using a kinetic modeling approach.

## Materials and Methods

### Materials

SP200 is an iron-reducing bacterium^9^ that was grown aerobically as batch cultures in LB medium to mid-exponential phase before use. Equine heart cytochrome c and 1,4-piperazinediethanesulfonic acid (PIPES) were purchased from Sigma Chemical Co. (China). K_2_Cr_2_O_7_ (99%) was purchased from Acros (USA) and was used without further purification.

### Quantification of *c*-Cyts in living SP200 cell suspension

The DT-UV/Vis spectra of the cell suspension were measured by a UV/Vis spectrophotometer (TU-1901 Beijing, China) equipped with an IS19-1 integrating sphere reflectance attachment. Using the spectral method, only the *c*-Cyts located on the very surface of cell outer-membrane can be measured directly, but the *c*-Cyts which located in periplasm and the cytoplasmic membranes were unlikely to be measured as the light was not able to penetrate the membrane. Hence, the *c*-Cyts measured in the cell suspension can only represent the outer membrane *c*-Cyts. According to Picardal *et al*.^9^, the *c*-Cyts were the important cytochromes present in the outer-membranes of SP200 with a heme-containing protein^8^. Here, SP200 was grown aerobically in nutrient broth at 30 °C for 18 h. Subsequently, the suspension was centrifuged at 7000 *g* for 10 min at 4 °C, and the pellets were washed three times and re-suspended in sterile PIPES buffer (pH 7.0) to an optical density of 1.2 (OD_600_), corresponding to approximately 1.5 × 10^11^ cells L^−1^. The calibration curve of hemes in *c*-Cyts of was obtained using horse heart cytochrome *c* with one heme in one molecule from the diffuse-transmittance UV/Vis spectra (Fig. S1a), and the slope of the calibration curve was observed as 25.68 mM^−1^ for reduced form (Fig. S1b). The actual *c*-Cyts concentrations in SP 200 suspension as indicated by the heme concentrations were calculated from slope value (25.68 mM^−1^).

### Cr(VI) reduction experiments

The intact SP200 cell suspension with an initial cell density of 1.5 × 10^11^ cells mL^−1^ was used for all the treatments in the Cr(VI) reduction experiments. Such a concentrated cell density of SP200 was used to shorten the reaction time (<1 h) and to reduce the interference of cell growth on the kinetic calculation; hence, the cell density can generally be assumed the same during the whole reaction period. The strain was aerobically inoculated in a nutrient broth for 16 h in a shaker at 180 rpm and 30 °C and harvested by centrifugation at 8,000 × g for 10 min at 4 °C for three times using PIPES buffer when it approached the exponential phase. The cell suspension in PIPES buffer was purged with 100% N_2_ for 30 min, and then the suspension with lactate (20 mM) as electron donor was added to a rectangular quartz cuvette with an optical path length of 1.0 cm for measurement before sealing in Anaerobic Chamber. Preliminary experiments showed that the *c*-Cyts was fully reduced in 1 to 2 minutes after the addition of lactate and hence, the initial *c*-Cyts was kept fully-reduced form in the anaerobic cuvette. The control without Cr(VI) was measured first, then Cr(VI) with different initial concentrations was added, and the spectra were taken at intervals. The scanning wavelength was from 300 nm to 700 nm. The specific peaks are 373 nm for Cr(VI) and 552 nm for reduced hemes. For the XPS analyses, the cells were exposed to 1000 μM Cr(VI) for 10 h under the optimal conditions. The cells were then separated by centrifugation at 10,000 × g at 4 °C for 5 min. The pellet was washed with deionized water and freeze-dried overnight in an oven at −42 °C.

### Method for extracting proteins containing OM *c*-Cyts

The extraction of proteins was performed by using one-step bacterium protein extraction reagent (BSP023, Sangong Biotech Co., Shanghai, China) containing 50 mM Tris-HCl, 2 mM EDTA, and 5% Triton X-100 at pH 8.5. The SP200 cell suspension was centrifuged at 7,000 g for 5 mins, and then the protein extraction reagent was added. After the mixture was centrifuged at 15,000 g for 5 mins, the liquid supernatant containing proteins was obtained. The obtained proteins contained fully-oxidized form of *c*-Cyts. The fully-reduced form of *c*-Cyts was prepared by adding excessive sodium ascorbate.

### Method for the 16S rRNA gene measurements

After around 60 mins of incubation, the cells was centrifuged at 7000 g for 4 min and quick-frozen by liquid nitrogen before the RNA being extracted by TRIzol reagent (Invitrogen, USA) and then reverse transcription PCR and Real-Time Quantitative PCR was used to test the amount of 16S rRNA gene. The obtained bacterial RNA was reverse transcribed using a PrimeScript II 1st strand cDNA Synthesis Kit (Takara, Shiga, Japan) according to the instructions of the manufacturer. Quantification of transcripts of bacterial 16S rRNA was determined by the iQ5 Real-Time PCR Detection System (Bio-Rid, USA) and using the SYBR Green I detection method. The qPCR System using the Eub338 (ACTCCTACG GGAGGCAGCAG)^32^ and Eub518 (ATTACCGCGGCTGCTGG)^33^ primer pair. Each 20 μL reaction solution contained the following: 1 μL of template cDNA (1–10 ng), 10 μL of 2 × IQ SYBR Green Supermix (Bio-Rid, USA), 0.2 μM of each primer. PCR conditions were 5 min at 95 °C, followed by 40 cycles of 94 °C for 20 s, 55 °C for 20 s, and 72 °C for 30 s^33^. Per DNA sample and the appropriate set of standards were run in triplicates. The qPCR calibration curves were generated with serial triplicate 10-fold dilutions of plamid DNA. The plamid pGEM-T Easy Vetor (Promega, Madison, USA) contained the cloned target sequences. Plasmid DNA was extracted using an EZNA Plasmid Mini Kit I (Omega Bio-Tek, Doraville, GA, USA) and the concentration was quantified by the Qubit 2.0 Fluorometer (Invitrogen, NY, USA). Target copy numbers for each reaction were calculated from the standard curves^34^.

### Numerical modeling

The kinetic model was fitted to the experimental data over a range of experimental conditions using the program Kintek Explorer^35^. To derive the optimal *k* values from the range of model fitting, the sensitivity of the model to changes in individual rate constant value was also assessed by examining the change in the relative difference between the experimental data and the kinetic model (defined as the relative residual, *r*) when one rate constant was varied while holding the others fixed at their optimal values. The program Kintecus^36^ was employed to evaluate the kinetic model using a Visual Basic for Applications (VBA) program to conduct the sensitivity analysis. Sensitivity analysis of the model was conducted by running the model whilst varying one rate constant with all other rate constants held constant at their optimized value, with the relative residual, *r*, determined over a range of values of each of the rate constants. The relative residual is defined as Eq. 1.


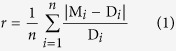


where M_*i*_ is the model response at a given system condition and time, D_*i*_ is the experimental data at the same given condition and time and *n* is the total number of measured data points over all conditions and times. The value of *r* represents the average deviation of the model result from the experimental data and is an indicator of the ability of the model to reproduce the experimental data.

## Results and Discussion

### Feasibility of *in Situ* monitoring Cr(VI) reduction by *c*-Cyts in living cell suspension

**Figure 1:**
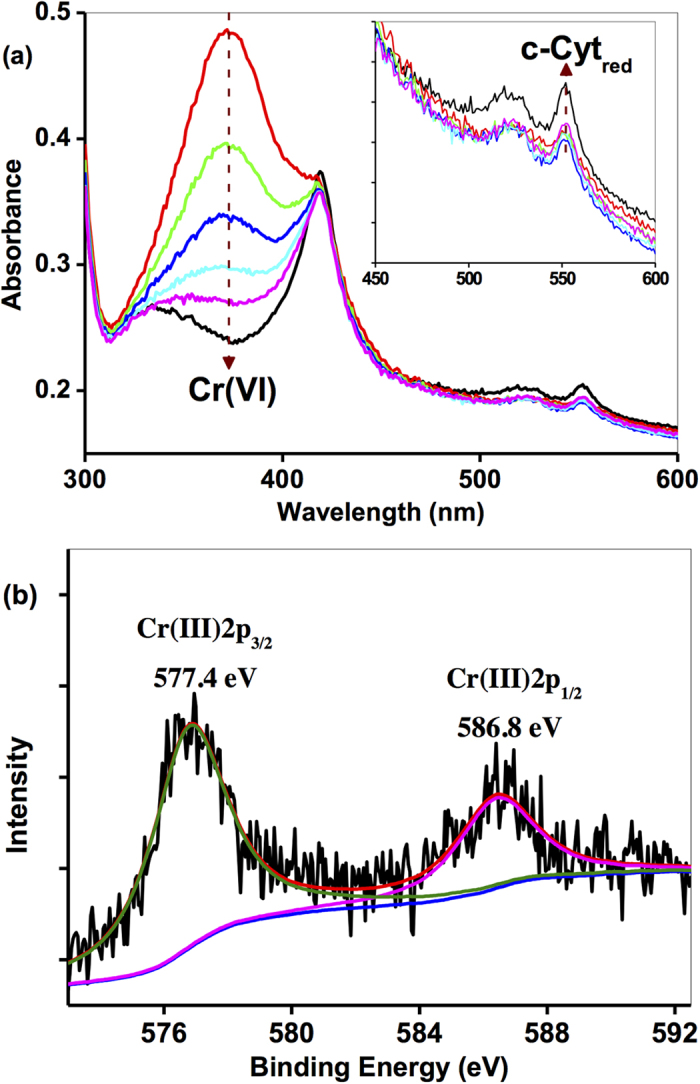
(**a**) The *in situ* kinetic spectra of intact SP200 cell suspensions incubated with 60 μM Cr(VI) under anoxic conditions at different time. Initial cell density of SP200: 1.5 × 10^11^ cells mL^−1^. The peak at 373 nm is attributed to Cr(VI), and the peak at 552 nm is attributed to Heme_red_ (insert). (**b**) XPS analysis of the Cr element in the sample after reaction.

The *in situ* kinetics of the reduction of Cr(VI) by *c*-Cyts in intact SP200 cells were conducted with 60 μM Cr(VI). The spectra in Fig. 1a showed that peaks at 373 nm, attributed to Cr(VI), decreased over time; simultaneously, the peaks at 552 nm, corresponding to reduced hemes (Heme_red_) in *c*-Cyts decreased as well. To further prove that the direct redox reaction between Cr(VI) and *c*-Cyts really occurred, the outer-membrane proteins have been extracted, and similar patterns of oxidized (*c*-Cyt_ox_) and reduced *c*-Cyts (*c*-Cyt_red_) were observed in the UV/Vis diffuse-transmittance spectra of whole cell and extracted OM proteins of SP200 (Fig. S2). After Cr(VI) was added to the reduced proteins containing *c*-Cyts, the peak intensity at 552 nm rapidly decreased, suggesting the fast oxidation of *c*-Cyt_red_ by Cr(VI). Even though the *c*-Cyts located in the periplasm and the cytoplasmic membrane might also be recorded in the spectra, with the addition of excessive Cr(VI), all the reduced *c*-Cyts in the liquid supernatant was completely oxidized, indicating that the reaction between *c*-Cyts and Cr(VI) definitely occurred. Therefore, this result can provide a direct evidence for the electron transfer from *c*-Cyt_red_ to Cr(VI).

Cr(III), as a well-recognized product of Cr(VI) reduction by DMRB^26^,^27^, was also found in the XPS test of sample after reaction in Fig. 1b, which was indicated by the peaks for Cr(III)2p_3/2_ at 577.4 eV and Cr(III)2p_1/2_ at 586.8 eV. A control in the presence of Cr(III) was also conducted with results in Fig. S3a suggesting that the reduction product (Cr(III)) did not interfere with the spectra for Cr(VI) or *c*-Cyts. In addition, a control with Cr(VI) and SP200 but without any lactate under oxic conditions (Fig. S3b) shows that there was no obvious reactivity for Cr(VI) by the oxidized hemes (Heme_ox_). Hence, it is feasible to directly measure Cr(VI) and Heme_red_ from the UV-Visible diffuse-transmittance spectra.

### Effect of Cr(VI) concentrations

As it has been widely reported that the high concentration Cr(VI) may induce the toxicity to the cells^28^,^29^,^30^,^31^,^37^, the alive cells were investigated through measuring the 16S rRNA of SP200 after incubation with Cr(VI) for 1 h. Results of 16S rRNA as a function of Cr(VI) (Fig. S4) show that while no obvious change on the number of 16S rRNA copies was observed in the presence of low concentrations of Cr(VI) (≤200 μM), the number of 16S rRNA copies decreased greatly when the Cr(VI) increased from 200 μM to 1000 μM. These results suggested that inhibitory effect on cell growth was very weak with Cr(VI) concentration less than 200 μM but occurred seriously in the presence of Cr(VI) with high concentrations >200 μM. As this study is aimed at disclosing the *in situ* reaction between Cr(VI) and *c*-Cyts in the living cells, the concentrations of Cr(VI) were chosen within a low range of 0–200 μM for the following studies to minimize the inhibitory effect of Cr(VI) on alive cell numbers.

Kinetics of Cr(VI) and *c*-Cyts were examined with different initial Cr(VI) concentrations from 0–160 μM (Fig. S5a). The pseudo-first-order rate constants (*k*) of Cr(VI) reduction were calculated as 0.708 min^−1^, 0.363 min^−1^, 0.127 min^−1^, and 0.065 min^−1^ with initial Cr(VI) concentrations 20 μM, 60 μM, 100 μM, and 160 μM, respectively, so the *k* values decreased with increasing initial Cr(VI) concentrations. The *c*-Cyt_red_ (Heme_red_) was measured as well with results in Fig. S5b that the Heme_red_ transiently dropped to a low level at the very beginning and then increased to different steady-state levels, which decreased in the same order as the increase of Cr(VI) concentrations from 0 to 160 μM.

### Kinetic models of Cr(VI) reduction by *c*-Cyts in intact cells

The experimental data in Fig. S5 were not able to be simply fitted using the typical model (e.g. pseudo-first order) due to the complexity of multi-reactants and multi-mechanisms. Previously, with other microorganisms, kinetic models for Cr(VI) reduction have been developed using a Michaelis-Menten approach based on the assumption of a single Cr(VI)-reducing enzyme^38^,^39^. Since Cr(VI) reduction in MR-1 does not appear to be the function of a single enzyme, the dual-enzyme model for Cr(VI) reduction by MR-1 was established by Viamajala *et al*.^40^ to describe the Cr(VI) reduction processes, which provided a tool to understand the enzyme-induced Cr(VI) processes, but the enzyme was not directly measured experimentally to further explore the enzymatic reactions. Hence, in this study, a model analysis was employed on a basis of the outer-membrane enzyme reaction mechanisms and the direct observation of spectral kinetics of Cr(VI) and *c*-Cyts. In the process of Cr(VI) reduction by *c*-Cyts of SP200, the electron transfer pathway can be concisely divided into two steps: (i) intracellular electron transport from electron donor to OM *c*-Cyts and (ii) electron transfer from *c*-Cyts to Cr(VI). The step-by-step potential losses can drive electron flow from electron donor via *c*-Cyts to Cr(VI), resulting in Cr(VI) reduction. While Cr(VI) can be reduced via both intracellular and extracellular ways^28^,^30^, the Cr(VI) reduction by the OM *c*-Cyts is more likely to occur due to the readily contact between Cr(VI) and OM *c*-Cyts before Cr(VI) diffusing into the periplasm of cells^27^,^28^ and hence, the extracellular Cr(VI) reduction by OM *c*-Cyts is supposed to be the dominant way of Cr(VI) reduction, which was also supported by the evidence of direct Cr(VI) reduction by extracted *c*-Cyts (Fig. S2). Therefore, the current study only focuses on the Cr(VI) reduction by the outer membrane *c*-Cyts. In Step (i), the electron donor (lactate) can be utilized by SP200 with concomitant intracellular electron transport from lactate to OM *c*-Cyts, resulting in the redox transformation of Heme_ox_ to Heme_red_ as shown in Eq. 2.


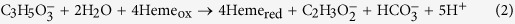


The Heme_ox_ and Heme_red_ represent the Fe(III)-associated and Fe(II)-associated hemes, respectively, hence the redox transformation from Heme_ox_ to Heme_red_ can be considered as an one-electron transfer reaction. As the concentration of lactate (20 mM) is much higher than that of the hemes (~1 μM) and the reaction is relatively fast (completed in an hour), the change in lactate concentration can be neglected, so Eq. 2 may be simplified to Eq. 3.


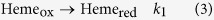


where *k*_1_ represents the first-order rate constant (s^−1^) of Eq. 3. The reaction rate (*r*_1_) can be expressed as Eq. 4.


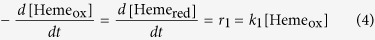


In Step (ii), Heme_red_ directly transfers electrons to Cr(VI) when Cr(VI) is in contact with the OM of SP200 as in Eq. 5. Under the circumneutral pH, the dominant species of Cr(VI) are CrO_4_^2−^ and HCrO_4_^−^, and the dominant aqueous species of Cr(III) include Cr(OH)^2+^, Cr(OH)_2_^+^, and Cr(OH)_3_(aq). Regardless of the different species, only total Cr(VI) and Cr(III) were taken into consideration to reduce the complication of models.


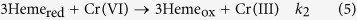


where *k*_2_ represents the second-order rate constant (M^−1^ s^−1^) of Eq. 5 by assuming that “3Heme_red_” is one molecule for convenience of the kinetic calculation. The reaction rate (*r*_2_) can be written as Eq. 6.


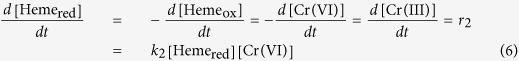


By combining Eqs 4 and 6, the reaction rate of hemes can be written as Eq. 7.


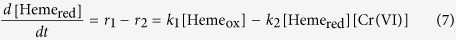


Based on Eqs 3 and 5, the kinetic model was established, and the experimental data for Cr(VI) and Heme_red_ were simulated with the result, as shown in Fig. S5, that *k*_1_ is 9.48 s^−1^ and *k*_2_ is 9.12 × 10^5^ M^−1^ s^−1^ for the hemes, equivalent to 9.12 × 10^4^ M^−1^ s^−1^ for the decaheme *c*-Cyts with ten hemes per protein, a value squarely between that of 3.5 × 10^4^ M^−1^ s^−1^ for MtrC and 2.5 × 10^5^ M^−1^ s^−1^ for OmcA^28^. While the fitting curves for Cr(VI) were matched with the experimental data, the fitting curves for Heme_red_ showed a marked discrepancy with the measured values. Particularly, while the fitting curves for Heme_red_ with 160 μM of Cr(VI) recovered to initial concentrations after 10 min, the experimental data dropped to considerably low, near-zero values. Further evidence for the constraint of various rate constants was obtained from the sensitivity analysis (Fig. S6). For Eq. 3, the best fitted values of *k*_1_ over considerable ranges (10^−3^–10^6^ s^−1^) had the residual value (*r*) 0.397, and the *r* value of the best fitted values of *k*_2_ from 10^4^ to 10^9^ M^−1^ s^−1^ for Eq. 5 was 0.429.

### Inhibitory effect of Cr(III) on Cr(VI) reduction and *c*-Cyts transformation

As the inhibition of cell growth in the presence of chromium has been widely observed^31^,^37^, Cr-induced inhibition to SP200 for Cr(VI) reduction must be considered here. The inhibitory effects of the reduction product, Cr(III), on bacterial cells were thought to contribute to this observed incomplete reduction of Cr(VI)^26^. The *c*-Cyt-mediated extracellular reduction of Cr(VI), coupled with subsequent extracellular precipitation of Cr(III), can serve as a mechanism for ameliorating the inhibitory effects of Cr(III) under Cr(VI)-reducing conditions, as the extracellular precipitation of Cr(III) on the cell surface occurred easily under the pH 7. Therefore, under low Cr(VI) concentrations, the Cr(III) played a dominant role of the inhibitory effect via the extracellular precipitation of Cr(III) on the cell surface, which was also consistent with that reported previously^26^.

**Figure 2:**
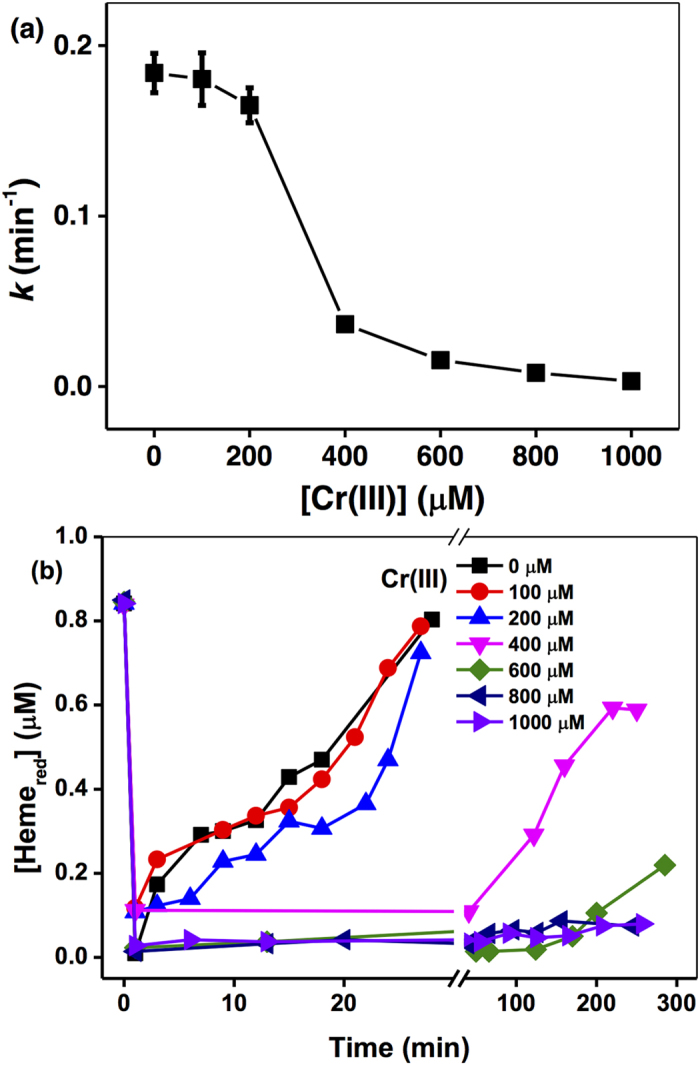
Cr(VI) reduction by *c*-Cyts in intact SP200 cell suspension in the presence of different [Cr(III)] from 0 μM to 1000 μM, (**a**) the pseudo-first-order rate constants (*k*) of the Cr(VI) reduction as a function of [Cr(III)], and (**b**) Heme_red_ transformation. All experiments were conducted with Cr(VI) 160 μM, SP200: 1.5 × 10^11^ cells mL^−1^.

To quantify the influence of Cr(III) on Cr(VI) reduction, different concentrations of Cr(III) from 100 μM to 1000 μM were added. Results in Fig. 2a suggested that, while the reaction rates of Cr(VI) reduction was just slightly affected by the addition of 100 μM as compared with control without Cr(III), the reduction rates of Cr(VI) decreased remarkably in the presence of increasing Cr(III) from 200 μM to 1000 μM, indicating that the Cr(III) might be harmful to SP200 as well. As shown in Fig. 2b, Heme_red_ in the presence of 100 μM Cr(III) was regenerated over time to the similar level as that without any Cr(III) addition, but failed in regeneration with Cr(III) concentrations of >400 μM. It was noted that the inhibitory effect of external Cr(III) was much weaker than that formed on cell with the equivalent Cr(III) concentrations, probably because Cr(III) is formed in the cell, while Cr(III) externally was not easily precipitated on the cell surface, resulting in the observed weak inhibitory effect of Cr(VI) reduction in Fig. 2. Based on the above results, it could be assumed that the Heme_ox_ might be combined with Cr(III) by forming unreactive hemes which can prevent the transformation from Heme_ox_ to Heme_red_, and finally inhibit the Cr(VI) reduction. Hence, the Cr(III)-induced deactivation from Heme_ox_ to Heme_red_ can be described as Eq. 8.


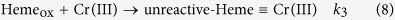


where unreactive-Heme ≡ Cr(III) represents the hemes lacking transformation into Heme_red_ after contact with Cr(III), and *k*_3_ represents the second-order rate constant (M^−1^ s^−1^) of Eq. 9 for Heme_ox_ inactivation by assuming the “3Heme_ox_” is one molecule for convenience of model calculation.


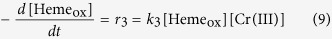


By combining Eqs 4, 6 and 9, the reaction rate of reactive Heme_ox_ can be written as Eq. 10.


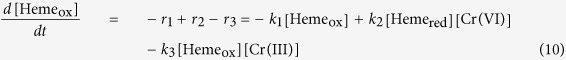


**Figure 3:**
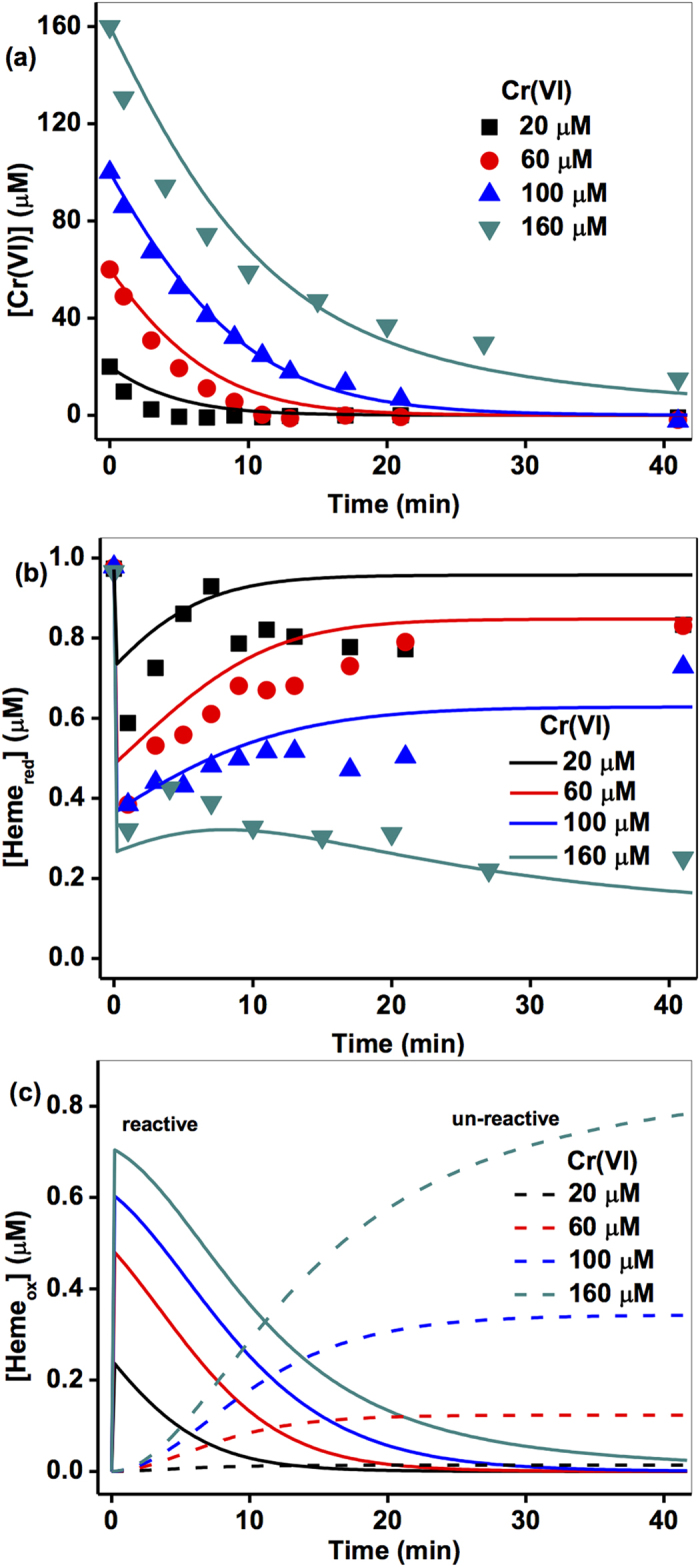
The kinetics of Cr(VI) reduction by *c*-Cyts in intact SP200 cell suspensions. (**a**) Cr(VI) reduction; (**b**) Heme_red_ reduction. Cr(VI): 20 μM–160 μM mg L^−1^, SP200: 1.5 × 10^11^ cells mL^−1^. (**c**) The predicted concentrations of reactive Heme_ox_ and un-reactive Heme_ox_. Solid lines represent the model fit using Eqs 2, 4 and 7 with rate constants *k*_1_ = 15.3 s^−1^, *k*_2_ = 7.68 × 10^4^ M^−1^ s^−1^ and *k*_3_ = 56.7 M^−1^ s^−1^.

Based on Eqs 3, 5 and 8, the kinetic model was revised, and the experimental data for Cr(VI) and Heme_red_ were simulated with results as shown in Fig. 3a,b, demonstrating that the fitting curves for Cr(VI) and Heme_red_ were better-matched with the experimental data, suggesting that (i) *c*-Cyt-mediated Cr(VI) reduction occurs and (ii) the reduction product Cr(III) is likely to inactivate the cellular effects of intracellular electron transport for Heme_red_ regeneration. Further evidence for the constraint of various rate constants was obtained from the sensitivity analysis (Fig. S7). For Eqs 3 and 5, the best fitted values of *k*_1_ (10^−3^–10^6^ s^−1^) and *k*_2_ (10^4^–10^9^ M^−1^ s^−1^) had the *r* values 0.206 and 0.207, respectively, which were both much lower than the *r* values (0.397 and 0.429) derived from the above two-reaction model, so this three-reaction model greatly improved the model fitting. In addition, the *r* value of the best fitted values of *k*_3_ from 10^4^ to 10^9^ M^−1^ s^−1^ for Eq. 5 was as low as 0.207, which can further highlight the important role of Eq. 8 in controlling the kinetics of Cr(VI) reduction by *c*-Cyts in the living cell suspension.

Although it is difficult to measure the dynamic changes of bacterial gene transcription related to the function of intracellular electron transport processes, the reactive Heme_ox_ and Cr(III)-induced un-reactive Heme_ox_ can be simulated based on the model established as in Fig. 3c, showing that the initial Heme_ox_ rose from zero to the maximum value in the first minute, accompanied by increasing Cr(VI) concentrations, and then decreased over time. Additionally, higher Cr(VI) concentrations led to a greater formation of un-reactive Heme_ox_, suggesting that the inhibition of Heme_red_ regeneration was strongly dependent on external Cr(VI) concentrations.

**Figure 4:**
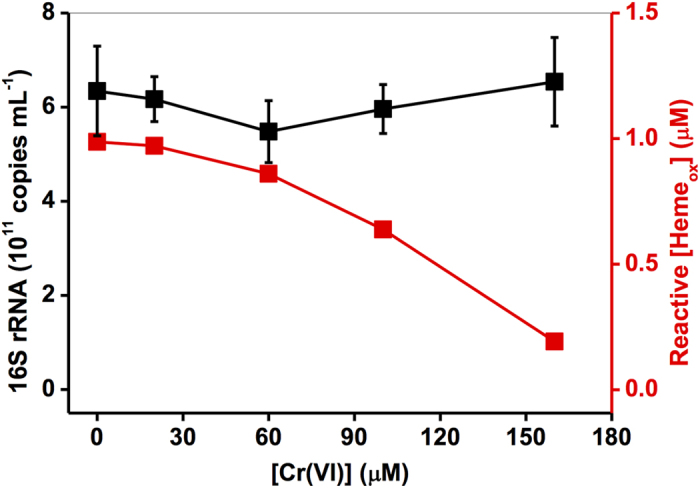
The 16S rRNA gene clone copies (black cubic-line) of SP200 (1.5 × 10^11^ cells mL^−1^) incubated in the presence of different concentrations of Cr(VI) for 40 min and the total reactive hemes (reactive [Heme_ox_]+[Heme_red_]) as a function of Cr(VI) concentrations. Cr(VI): 20 μM – 160 μM (red cubic-line). Error bars represents the standard deviation of the mean (n = 3).

The effect of chromium on the growth of SP200 was investigated by examining 16S rRNA after incubation of SP200 with Cr(VI) for 40 min. Fig. 4a shows that just a slight change on the number of 16S rRNA copies was observed in the presence of low concentrations of Cr(VI) (≤160 μM). For comparison, the total concentration of reactive hemes, calculated by combining the model values of Heme_red_ (Fig. 3b) and reactive Heme_ox_ (Fig. 3c) at 40 min, was also plotted as a function of Cr(VI) concentration in Fig. 4, showing that an evident reduction of reactive hemes was caused by Cr(VI) even with concentrations ≤160 μM. The inconsistency between reactive hemes and 16 S rRNA under Cr(VI) concentrations ≤160 μM indicated that the cellular function of redox transformation of Heme_ox_ to Heme_red_ might readily be inhibited by low concentrations of Cr(VI), while the inactivation of bacteria only occurred under high Cr concentrations. Therefore, the *c*-Cyt-mediated Cr(VI) reduction and Cr(III)-induced inhibitory effects on the redox transformation of hemes were well-described by the model as established by Eqs 3, 5 and 8.

### Significance and application of the established kinetic model

While the *c*-Cyts are only present in the low concentrations in μM in the OM of SP200, the direct and accurate observation of absorbance changes in intact organisms is a useful complement to traditional reductionist approaches and recent advances in proteomic and transcriptomic studies^19^. Given that the transient changes of the redox state of hemes can be monitored with great sensitivity by the simple spectrometric method, this study under non-invasive physiological conditions provides a suitable and powerful approach to examine the extent and rates of biological events *in situ* without disrupting the complexity of the live cellular environment as it occurs in the intact bacterium. Here, the advantages of the *in situ* spectrophotometric approach are highlighted by monitoring Cr(VI) and *c*-Cyts in intact SP200 cell suspensions simultaneously.

As *c*-Cyts play key roles in extracellular electron transfer processes^11^,^12^,^13^ the direct measurement of *c*-Cyts reflects the real physiological and metabolic functions that take place during extracellular Cr(VI) reduction^13^. For example, as the concentration of Cr(VI) rises, the regeneration rate of the *c*-Cyts from the oxidized form to reduced form is lowered, indicating that the inhibitory effects of Cr(VI) induced the loss of this cellular function. While toxicity studies are usually conducted by the traditional approach^26^,^27^,^28^,^29^,^30^,^31^,^37^, this study suggests that the *c*-Cyts obtained from the *in situ* spectrophotometric approach can be used as an indicator for evaluating the inhibitory effects of other substrates on the physiological and metabolic functions of DMRB. Although the *in vitro* reaction kinetics between outer-membrane enzymes and Cr(VI) were examined with purified proteins^13^,^28^ the other reactions involved in the electron transfer chain were not taken into consideration in such *in vitro* studies. The *in vivo* kinetics and application of modeling approach can give the rate constant between *c*-Cyts and Cr(VI) in a living cell suspension, and besides, the recovery rate of reduced hemes and the complexation rate between Cr(III) and hemes can also be taken into account to illustrate the electron transfer processes.

In addition, by employing the model approach, the detailed kinetics of *c*-Cyts in redox states and cellular functions were readily obtained, and, hence, the changes in the thermodynamic properties of *c*-Cyts in living cells can also be obtained. In terms of the Nernst Equation for *c*-Cyts, an attempt has been made to calculate the actual redox potential of *c*-Cyts in living SP200 cells with Cr(VI). The electrons are released from the outer membrane (OM) of the cell, through OM *c*-type cytochromes (*c*-Cyts), into the extracellular electron transfer mechanism and hence, the Heme_ox_ in oxidized *c*-Cyts are transformed into the Heme_red_ in the reduced *c*-Cyts via the reduction by electrons. The half-cell reaction is written as Eq. 11.


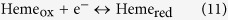


**Figure 5:**
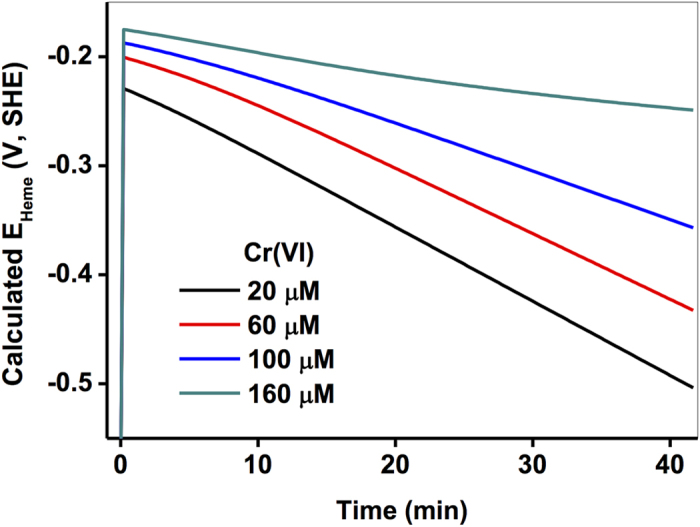
Calculated specific redox potentials of *E*_Heme_ of SP200 with 20 μM – 160 μM mg L^−1^ of Cr(VI). The solid lines were calculated from fitting curves for Heme_red_ in Fig. 3b and reactive Heme_ox_ in Fig. 3c based on Eq. 11.

The specific redox potentials of *c*-Cyts can be calculated by the Nernst equation (Eq. 12).


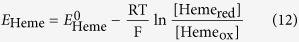


where 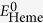 represents the standard redox potentials of hemes, R is the ideal gas constant (8.3145 J mol^−1^ K^−1^), F is the Faraday constant (96,485 C mol^−1^ e^−^), and T is the temperature (298 K). Because the values of 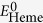 were reported previously in a wide range of −0.4 V–0 V^7^,^41^,^42^ an average value of −0.2 V (vs. SHE) was used here for the calculation of specific *E* values (*E*_Heme_). Results in Fig. 5 show that the *E*_Heme_ of SP200 became progressively more positive with an increase in Cr(VI) concentrations, indicating that the extracellular electron transfer capacities of OM *c*-Cyts in SP 200 decreased due to the inhibitory effects of Cr on the regeneration of reduced *c*-Cyts via intracellular electron transport. Therefore, this study will have substantial implications for disclosing the roles of *c*-Cyts in intact cells on the Cr(VI) reduction from both kinetics and thermodynamics. Despite the recent progress in describing the *c*-Cyt-mediated Cr(VI) reduction by a concise model, we still face major challenges in unraveling and understanding the intricate and coupled physiological processes that control metabolisms and electron transport, and the correlation of kinetics with genetic information (DNA, RNA, etc.) would be helpful and are indeed necessary for future work on modeling microbial metal reduction processes.

In conclusion, the kinetics of Cr(VI) reduction by *c*-Cyts in living *Shewanella putrefaciens* 200 cell suspension were successfully. The Cr(VI) reduction rates decreased with the increase of Cr(VI) concentrations, which might be attributed to the inhibitory effect of Cr(III) from the 16S rRNA analysis and the experiments with added Cr(III). A brief kinetic model was established with three predominant reactions, redox transformation of *c*-Cyts, low concentration Cr(VI) reduction by *c*-Cyt_red_, and the Cr(III)-induced inhibition of heme redox transformation, resulting in successfully fitting the experimental data. While the low concentration Cr(VI) could not kill the bacteria directly indicated by 16S rRNA analysis, the Cr-induced inhibitory effect to redox transformation of *c*-Cyts was observed. This study may be very helpful for interpreting the *in vivo* enzymatic metal reduction from a kinetic perspective of key electron transfer proteins.

## Additional Information

**How to cite this article**: Liu, T. *et al*. *In Situ* Spectral Kinetics of Cr(VI) Reduction by *c*-Type Cytochromes in A Suspension of Living *Shewanella putrefaciens* 200. *Sci. Rep.* **6**, 29592; doi: 10.1038/srep29592 (2016).

## Supplementary Material

Supplementary Information
