# Supplementary material for: In Situ Spectral Kinetics of Cr(VI) Reduction by c-Type Cytochromes in A Suspension of Living Shewanella putrefaciens 200
Source: Sci Rep. 2016 Jul 11;6:29592. doi: 10.1038/srep29592 (PMC4939527; doi:10.1038/srep29592)
Supplement: Supplementary Information [file srep29592-s1.pdf]

Supporting information

In Situ Spectral Kinetics of Cr(VI) Reduction by *c*-Type Cytochromes in A Suspension of Living *Shewanella putrefaciens* 200

Tongxu Liu[1], Xiaomin Li[1,2], Fangbai Li[1*], Rui Han[1], Yundang Wu[1], Xiu Yuan[2] & Ying Wang[1]

[1]*Guangdong Key Laboratory of Agricultural Environment Pollution Integrated Control, Guangdong Institute of Eco-Environmental and Soil Sciences, Guangzhou, P. R. China 510650.* [2]*School of Civil and Environmental Engineering, University of New South Wales, Sydney, NSW, Australia 2052. Correspondence and requests for materials should be addressed to F.B.L. (email: cefbi@soil.gd.cn)*

Figure S1

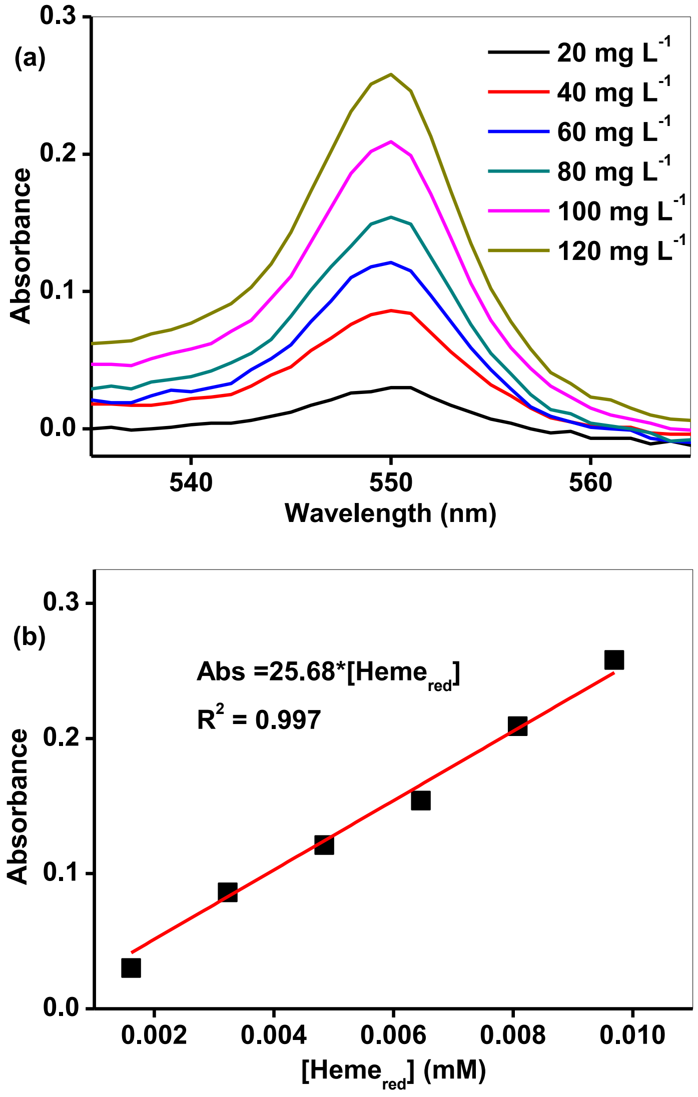

Figure S1. The diffuse-transmittance UV-Vis spectra of cytochrome *c* from equine heart (Sigma, C2506) with different concentrations (20 mg L^{-1}, 40 mg L^{-1}, 60 mg L^{-1}, 80 mg L^{-1}, 100 mg L^{-1}, 120 mg L^{-1}) (a) and the peaks at 550 nm as a function of the $Heme_{red}$ concentrations (b). According to the calibration of results obtained at 550 nm, the slope was calculated as 25.68 mM^{-1}, which was used to calculate the *c*-Cyt concentrations in living cells. Since the cuvette used in this measurement had 1 cm light path, the ε_{550nm} was calculated as 25680 M^{-1} cm^{-1}, which was a little lower than the reported value (29500 M^{-1} cm^{-1}) (Taniguchi et al., 1984; Vanderkooi et al., 1976).

Taniguchi I., Iseki M., Toyosawa K., Yamaguchi H., Yasukouchi K. Purines as new promoters for the voltammetric response of horse heart cytochrome *c* at a gold electrode. *J. Electroanal Chem.* (1984) 164, 385-391.

Vanderkooi J.M., Adar F., Erecińska M. Metallocytochromes *c*: Characterization of electronic absorption and emission spectra of Sn^{4+} and Zn^{2+} cytochromes *c*. *Eur. J. Biochem.* (1976) 64, 381-387.

Figure S2

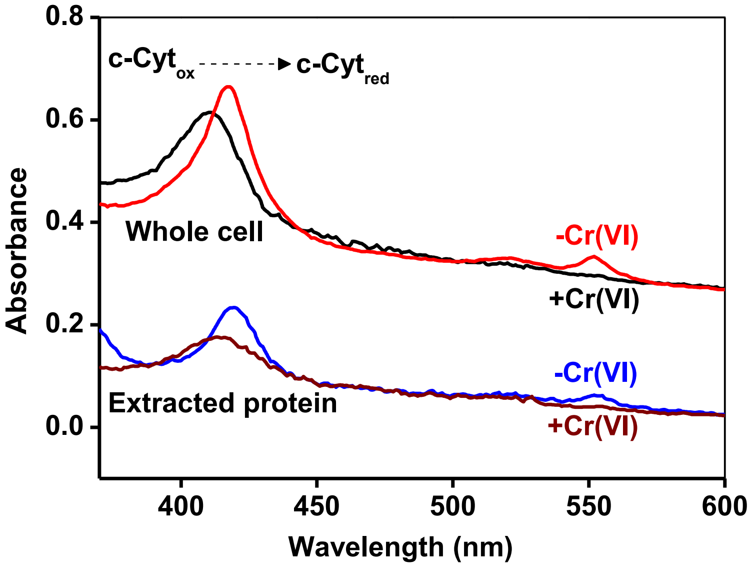

Figure S2. The UV/Vis diffuse-transmittance spectra of whole cells and extracted proteins of SP200 before and after addition of Cr(VI) (50 μM). Cell density of SP200: 1.5×10^{11} cells mL^{-1}.

Figure S3

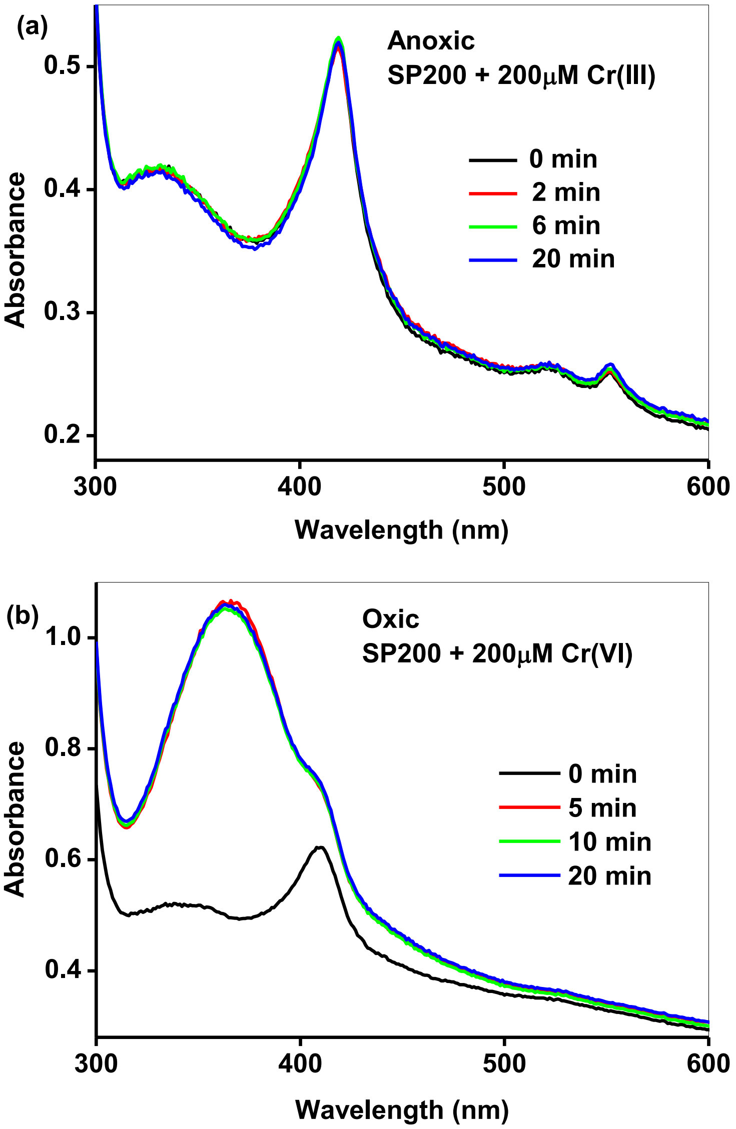

Figure S3. The kinetic spectra of (a) intact SP200 cell suspensions with Cr(III) (200 μM) incubated under anoxic conditions at different times and (b) intact SP200 cell suspensions with Cr(VI) (200 μM) incubated under oxic conditions at different times. Cell density of SP200: 1.5×10^{11} cells mL^{-1}.

Figure S4

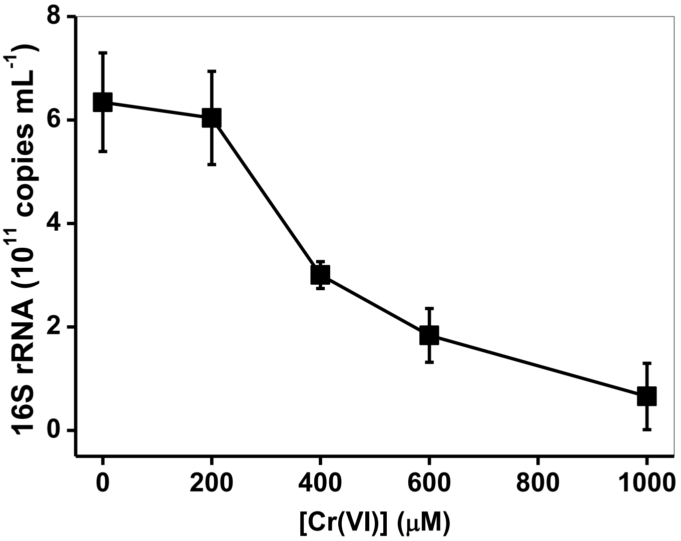

Figure S4. The 16S rRNA gene clone copies of SP200 incubated after 1 h as a function of [Cr(VI)] from 0 μM to 1000 μM. Initial cell density of SP200: 1.5×10^{11} cells mL^{-1}.

Figure S5

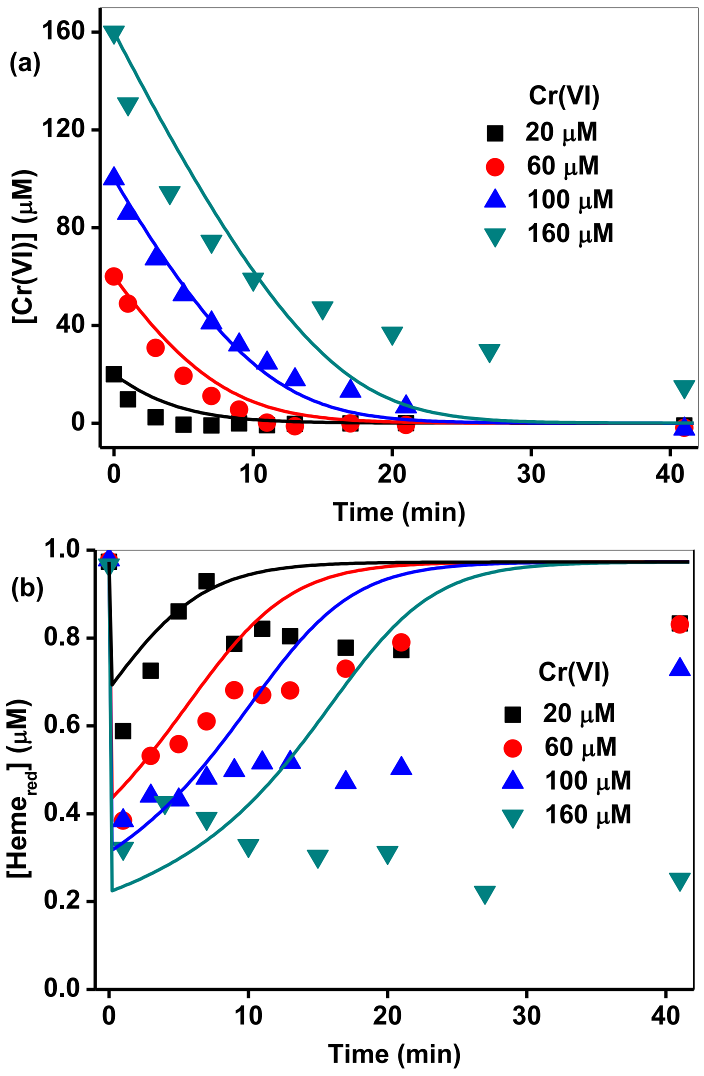

Figure S5. The kinetics of Cr(VI) reduction by *c*-Cyts in intact SP200 cell suspensions. (a) Cr(VI) reduction; (b) $Heme_{red}$ transformation. Cr(VI): 20 μM – 160 μM mg L^{-1}, SP200: 1.5×10^{11} cells mL^{-1}. Solid lines represent the model fit using Eqs. 2 and 4 with rate constants k_1 = 9.48 s^{-1} and k_2 = 9.12×10^{5} M^{-1} s^{-1}.

Figure S6

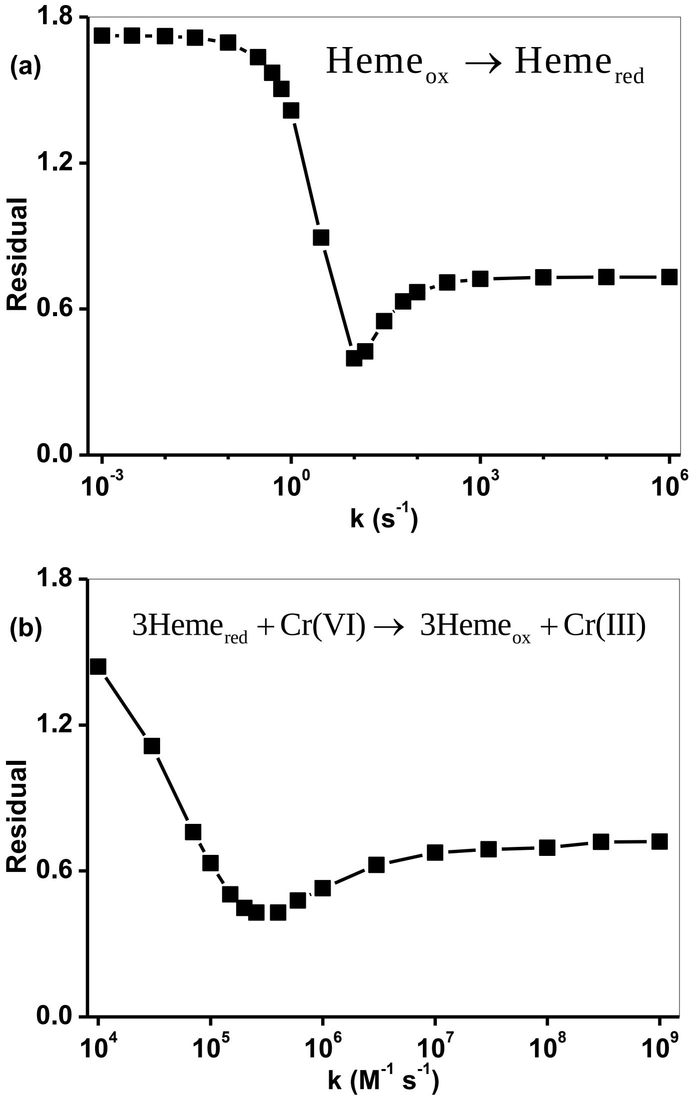

Figure S6. Sensitivity analysis for reaction rate constants (Eqs. 2 and 4).

Figure S7

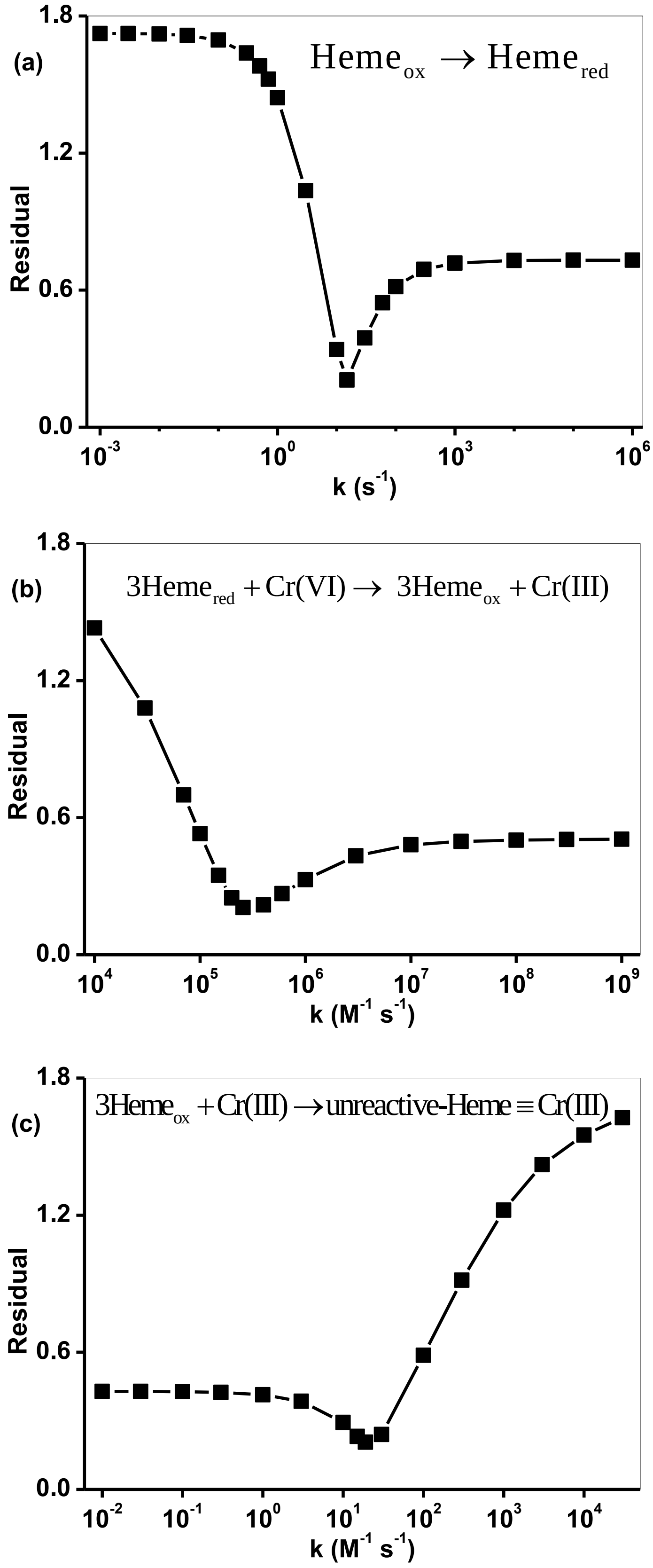

Figure S7. Sensitivity analysis for reaction rate constants (Eqs. 2, 4, and 7).
